# Accuracy of the revised Addenbrooke Cognitive Examination (ACE-R) and Mini-Mental (MMSE) in a Quilombola community with low education attainment: results of a cross-sectional study

**DOI:** 10.3389/frdem.2025.1673362

**Published:** 2026-02-02

**Authors:** João de Deus Cabral Júnior, Bruno Luciano Carneiro Alves de Oliveira, Sharon Sanz Simon, Jhule Silva Passinho, Carolina Cappi, Laiss Bertola, Candida Helena L. Alves, Vanda M. F. Simões, Gilberto Sousa Alves

**Affiliations:** 1 Postgraduate Program in Health Sciences (PPGCS), Federal University of Maranhão, São Luís, Brazil; 2 Department of Public Health, Federal University of Maranhão, São Luís, Brazil; 3 Krieger Klein Alzheimer’s Research Center, Rutgers University, Newark, NJ, United States; 4 Department of Psychiatry, New Jersey Medical School, Rutgers University, Newark, NJ, United States; 5 CARE - Research Center on Health and Social Sciences, Portalegre, Portugal; 6 Department of Psychiatry, Icahn School of Medicine at Mount Sinai, New York, NY, United States; 7 Geriatrics Service, Faculty of Medicine, University of São Paulo, São Paulo, Brazil; 8 Department of Psychology - Faculty of Medical Sciences of Minas Gerais, Belo Horizonte, Brazil; 9 Translational Psychiatry Research Group, Federal University of Maranhão, São Luís, Brazil; 10 Postgraduate Program in Psychiatry and Mental Health (PROPSAM), Institute of Psychiatry of the Federal University of Rio de Janeiro, Rio de Janeiro, Brazil

**Keywords:** mild cognitive impairment, cognitive screening, quilombolas, illiterate, Addenbrooke, accuracy, Mini-Mental State Exam

## Abstract

**Background:**

Illiteracy may overestimate screening test interpretation for older adults with suspected cognitive decline. In rural areas of Brazil, the illiteracy rate and the lack of valid cognitive instruments may postpone the diagnosis of cognitive disorders.

**Objective:**

To determine the diagnostic accuracy of the Addenbrooke’s Cognitive Examination (ACE-R) and Mini-Mental State Exam (MMSE) as well as the prevalence of mild cognitive impairment (MCI) for older adults with low education in a Quilombola community placed in Northeast Brazil, compared to cognitively healthy.

**Methods:**

204 participants were collected from a sociodemographic and clinical evaluation and were subsequently the ACE-R and MMSE. Among them, 25 subjects presented MCI, and 179 were classified as cognitively healthy.

**Results:**

The prevalence of MCI was 12.3%; optimal accuracy yielded highest values for the comparison between controls and MCI for both ACE-R [Area Under the Curve (AUC) = 0.96] and MMSE (AUC = 0.96).

**Conclusion:**

These findings support establishing reliable cutoff scores for cognitive assessment of older adults with low educational attainment, living in rural areas.

## Introduction

The increase in life expectancy in Brazil has been potentially associated with a higher prevalence of age-related disorders and cognitive decline (Herrera et al., 2002; Chaves and Chaves, 2014). According to the World Health Organization of the United Nations (WHO-UN), Brazil is one of 10 countries with the world’s largest population of older adults (World Health Organization, 2021). Data from the Brazilian Institute of Geography and Statistics (IBGE) show that older adults aged 65 or older represented 10.9% of the total population in 2022 (Instituto Brasileiro de Geografia e Estatística, 2022), an increase of 57.4% from the previous Census and a growth rate nine times higher than the global population (2010) (Instituto Brasileiro de Geografia e Estatística, 2010), with an impact on the population aging index, which in 2022 reached 55.2 (Instituto Brasileiro de Geografia e Estatística, 2022). Population aging is accompanied by greater dependency and disability among individuals over the age of 65 and holds substantial public health challenges (World Health Organization, 2021; Instituto Brasileiro de Geografia e Estatística, 2022). According to the 2022 Census Panorama (Instituto Brasileiro de Geografia e Estatística, 2022), the percentage of people living in rural areas in the country is 12.59%. In Maranhão, this proportion accounts for 29.07% of the total population, making it the second most representative state in Brazil (Instituto Brasileiro de Geografia e Estatística, 2022).

Rural populations are often less studied in terms of their health behaviors, associated risks, and social, racial, and community determinants of their physical and mental health (Barros and Goldbaum, 2019; Oliveira and Pinheiro, 2023). These demands become even more critical in minoritized populations and those of African ancestry in the country, such as the Quilombolas. Quilombolas are descendants of enslaved people who sought refuge and remained in rural and remote communities to escape the enslaved Brazilian and racist system that prevailed for almost four of the five centuries of Brazil’s existence (Bohn and Grossi, 2018; Silberling, 2003). They are present throughout Brazil, representing 0.66% of the total population; however, in the state of Maranhão, this proportion rises to 3.97%, making it the second most populous state among both Quilombola populations (269,074) and the number of communities (27.7%) (Instituto Brasileiro de Geografia e Estatística, 2022). The Quilombola population living in rural areas represents 61.71%, with the Northeast region having the lowest percentage of this group living in urban areas in Brazil (Instituto Brasileiro de Geografia e Estatística, 2022).

Their territories are characterized by vulnerability, material and social deprivation, and a high need for access to health, education, and basic sanitation (Macedo Pires Ferreira et al., 2020). The accumulation of public disadvantages among Quilombola communities has led to lifestyle vulnerability potentially associated with age-related cognitive disorders. In Brazil, another relevant risk factor for cognitive disorder, low education, stands as the most significant population-attributable fraction (PAF) to prevent dementia risk, around 9.5% according to recent evidence (Suemoto et al., 2025). According to IBGE, the illiteracy rate among older adults can be up to three times higher than that of individuals with 15 years of formal education or more, currently reaching 16% (Instituto Brasileiro de Geografia e Estatística, 2022). Among Quilombolas individuals aged 65 and older, the illiteracy rate is 53.93%, significantly surpassing the illiteracy rate among Black and Brown individuals in the Northeast, which stands at 30.7% (Instituto Brasileiro de Geografia e Estatística, 2022). As education plays a crucial role in cognitive resilience (Carret et al., 2005; Hall et al., 2007), addressing this factor could have substantial implications for dementia prevention strategies (Sisco et al., 2015).

A critical topic that is often overlooked is how cognitive performance among illiterate individuals is interpreted, considering Brazil’s diverse geographic and cultural contexts. In socioeconomically less developed regions, such as the Brazilian Northeast, intra-regional disparities in access to healthcare, particularly between urban and rural areas, alongside increased exposure to risk factors, may increase the risk of cognitive decline in the latter (Livingston et al., 2024; Suemoto et al., 2023). In fact, rural populations tend to have higher rates of poverty, lower access to basic and specialized health services, and greater exposure to risk factors such as depression and cardiovascular disease (Mfene and Pillay, 2024). Therefore, whether rural populations have been more vulnerable to developing mild cognitive impairment (MCI) than urban populations is a matter of debate (Livingston et al., 2024; Suemoto et al., 2023). Like any other population, preventive approaches in rural communities should be driven to identify general risk factors, ideally at an early stage of life, and screen for individuals at higher risk for dementia, e.g., MCI (Livingston et al., 2024; Suemoto et al., 2023); like mentioned by the Lancet Commission, it is never too early or too late to reduce dementia risk (Livingston et al., 2024). Thus, rural-based older Quilombolas characterize a typically underserved population at high risk for cognitive disorders in aging, but remain underrepresented in health research.

Another relevant aspect is the definition of cognitive impairment non-dementia (CIND). The concept of CIND is a broader definition of impairment encompassing individuals who meet the criteria for MCI as well as others who are cognitively impaired but do not fulfill all the criteria for MCI (Unverzagt et al., 2001; Petersen et al., 2009). MCI, conversely, requires cognitive complaint, cognitive decline, or impairment, objective evidence of impairment in cognitive domains, essentially normal functional activities, and absence of dementia (Unverzagt et al., 2001; Petersen et al., 2009). MCI is deemed a clinical intermediate stage between healthy aging and pathological aging (Petersen et al., 2009; Petersen, 2004; Albert et al., 2011; Scazufca et al., 2009). The worldwide prevalence of MCI in people over 65 years of age is 12–18% (Unverzagt et al., 2001); around 50% of MCI subjects may develop dementia within 3 years, and from baseline (MCI diagnosis), the annual conversion to dementia (all types) ranges from 6–15% (Breton et al., 2019; Tierney et al., 1996; Mitchell and Shiri-Feshki, 2009). In Brazil, the incidence rate of MCI ranges from 6.1 to 13.2% (Chaves et al., 2009; Godinho et al., 2012), although estimations based on population studies in Northeast Brazil are scarce.

Cognitive tests are commonly used to screen cognitive impairment, diagnose etiologically, establish disease severity, and monitor disease progression (Robert et al., 2003). A significant challenge in the initial assessment of age-related cognitive disorders is selecting a screening test that is both sensitive and specific for differential diagnosis. For adults who are either illiterate or have low levels of education, additional challenges are posed in cognitive assessment. Previous studies have sought to establish valid cutoff scores for illiterate adults (Herrera et al., 2002; Chaves and Chaves, 2014), but there is no consensus on whether data can be replicable in populations from different regions nationwide. For most old-age public services in Brazil, complete neuropsychological batteries are unavailable. Therefore, reliable, rapid, and user-friendly tools are necessary to accurately screen for MCI.

The Brazilian Health Ministry has recently issued general guidelines for screening cognitive decline in the community. Although newer instruments, such as the 10-Point Cognitive Screener (10-CS) and the Brief Cognitive Screening Battery (BCSB), have been officially recognized as valuable, the ecological validity and accuracy of these instruments in diverse regions across Brazil require further evidence. Conversely, standard instruments for briefly screening cognitive decline in illiterates, such as the Mental State Exam (MMSE) and the Addenbrooke’s Cognitive Examination-Revised (ACE-R), are deemed relevant in general practice and specialized services and are officially recommended by the Brazilian Academy of Neurology in 2022 (Smid et al., 2022). Indeed, most of accuracy studies employing ACE-R and MMSE were conducted with wealthier, urban population, outside thus rural zones; experts, notably for the MMSE have acknowledged the influence of educational background (Smid et al., 2022; Bertola et al., 2019). Brucki et al. (2003) reported that educational level was the most critical factor influencing MMSE scores (ANOVA F [4, 425] 100.45, *p* < 0.0001), and cutoff scores for illiterate people, based on median values (score = 20), were the lowest across all groups evaluated.

Evidence has also indicated ACE-R as a valuable tool for differentiating between healthy controls and MCI (Alexopoulos et al., 2010; Lonie et al., 2010). ACE-R was initially validated in 2012 by Amaral-Carvalho and colleagues and contains suggestions for data interpretation according to literacy level (Petersen, 2004). A greater diagnostic accuracy of the ACE-R compared to the MMSE, with less educational bias and broader cognitive evaluation, has been suggested (Larner and Mitchell, 2014). Two previous studies of our group, both conducted with outpatient subjects living in urban zones of Northeast Brazil, have identified lower scores for ACE-R and MMSE in the performance of illiterate and low schooling participants (either MCI or healthy controls) when compared to other similar Brazilian studies; for a thorough review, see Tavares-Júnior et al. (2021) and Passinho et al. (2024).

The study of psychometric properties of ACE-R and MMSE in underserved regions, such as the Quilombola rural communities of Northeast Brazil, through the estimation of optimal cutoff points for cognitive screening, is of paramount importance. First, it will help reduce the educational bias typically seen in cognitive scales validated for the Brazilian population, thereby reducing the risk of false positives. Secondly, it may contribute to the early detection of MCI and the secondary prevention of dementia cases, making their use practical and adapted to local reality (Caldas et al., 2012). Our study aimed, thus, to determine the diagnostic accuracy of the ACE-R and MMSE as cognitive screening tools for older adults with low levels of education and non-dementia status living in a rural area in Northeast Brazil. Our primary objectives stand as follows: (1) to determine the prevalence of MCI within the Quilombola community; (2) to establish the accuracy of the ACE-R and MMSE tests in distinguishing between healthy individuals and MCI, comparing these results with other population-based studies with similar characteristics.

## Methods

### Participants

**Figure 1:**
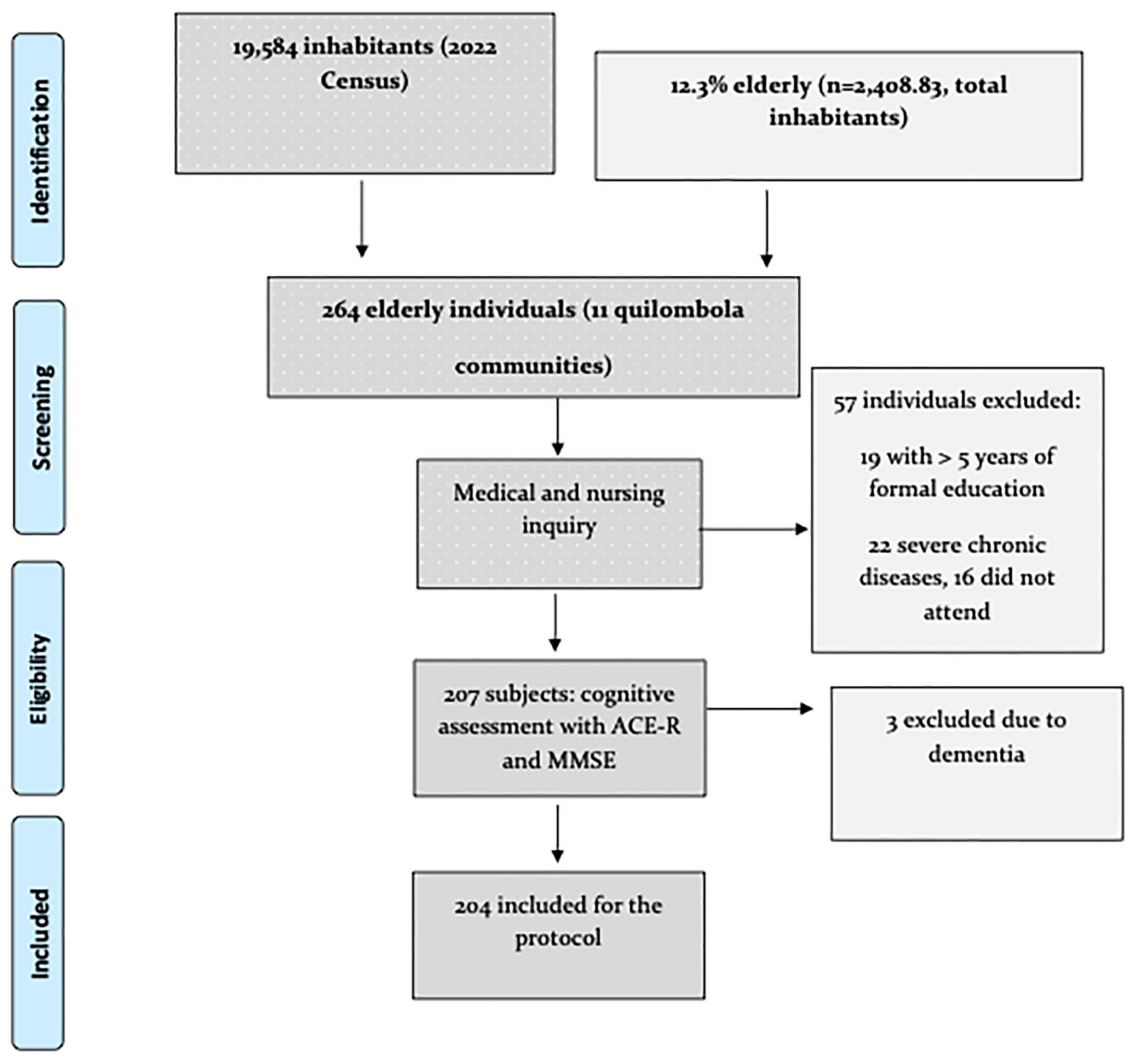
Flowchart depicting the study design.

This current investigation is part of the Population Survey of the Living Condition and Health Status of Older Persons in Quilombola Communities (IQUIBEQ) project, placed in the Baixada Maranhense with local services and health workers (Barros et al., 2023). The cross-sectional study of the household-based population survey type is linked to a larger research project entitled “Conditions of Life and Health of the Elderly Quilombolas of a City of Baixada Maranhense,” a branch of the IQUIBEQ project. In 2022, the total area of the municipality of Bequimão was 790,222 km^2^, and the census population was 19,584 inhabitants (12.3% elderly). The Human Development Index (HDI) was 0.60, and the Gross Domestic Product (GDP) per capita was R$1,253.43 ($ 241,04). Hence, we were faced with a local population of 264 older adults, and after screening procedures, the total number of Quilombola older adults for analysis remained at 204. The sample identification and selection methodology is demonstrated in Figure 1.

### Experimental procedures

The IQUIBEQ study protocol included a population, household-based cohort of subjects aged 60 or older living in 11 rural communities within the municipality of Bequimão, Maranhão State, northeast Brazil. The Palmares Cultural Foundation and the Brazilian Ministry officially recognize these communities as descendants of the original Quilombola population (Barros et al., 2023). Data collection took place between September 2022 and March 2024.

A comprehensive evaluation, encompassing sociodemographic characteristics, living conditions, physical health, psychiatric symptoms, and cognitive functioning, was conducted by trained interviewers under the supervision of a geriatric psychiatrist, a senior neuropsychologist, and a senior nurse. According to the flowchart, all individuals aged 60 or older were interviewed alongside their family members and were screened and submitted for further evaluation. Major psychiatric disorder (bipolar disease, schizophrenia) or any severe medical condition were also considered exclusion criteria. Low education was defined as having up to 5 years of formal education, as reported by the subject and verified by family members.

To exclude the diagnosis of dementia, DSM-5 (American Psychiatric Association, 2022), and International Statistical Classification of Diseases and Related Health Problems (ICD-10) (World Health Organization, 1992) criteria were employed. All subjects and their family members were evaluated using a clinical interview questionnaire, MMSE, and the Brazilian-validated version of the Clinical Dementia Rating (CDR) (Hughes et al., 1982; Fagundes Chaves et al., 2007) and Pfeffer Functional Activities Questionnaire (FAQ) (Pfeffer et al., 1982; Assis et al., 2014) to assess cognitive status and functional ability. The level of depressive symptoms was rated by the 15-item Geriatric Depression Scale (GDS) (Yesavage et al., 1982; Dias et al., 2017).

#### Group definition

MCI diagnosis was based on Petersen criteria and included the following characteristics: (1) cognitive complaints reported by either the patient or informant; (2) history of cognitive decline during the past year; (3) cognitive changes (memory and/or other domains) observed at the clinical objective evaluation, comparatively with normal adults of the same age and educational level; (4) absence or minimal difficulty with daily activities, preserved general cognitive functioning; and (5) absence of dementia. To fulfill item (3) and determine MCI status, we adapted from Brucki and Nitrini (2010) weighted scores (*z* scores) of MMSE subsets (Petersen, 2004); patients were labeled MCI if any cognitive domain was (were) lower than 1.5 SD (Petersen, 2004), and if CDR was equal to 0.5. To define the absence of difficulty with daily activities, we considered participants showing FAQ (Pfeffer et al., 1982; Assis et al., 2014) scores lower than 5, as the Brazilian Academy of Neurology recommended (Smid et al., 2022). Healthy controls were defined if no criteria for MCI were fulfilled and CDR was equal to 0. All subjects considered eligible for the study were carried out with the ACE-R.

#### Exclusion criteria

The main exclusion criteria were dementia, delirium (confusional state), patients unable, for any reason, to respond to the interview, history of stroke, report of epilepsy, severe neurological disorder (e.g., traumatic brain injury), deficit in hearing or visual impairment, major depression episode, bipolar disorder, schizophrenia, or alcohol dependence. The clinical exam was performed for all subjects and included a psychiatric interview based on the Diagnostic and Statistical Manual of Mental Disorders (DSM-5) (American Psychiatric Association, 2022). Furthermore, blood tests were performed if necessary to rule out medical causes of cognitive impairment.

### Ethical statement

The study was approved by the National Research Ethics Committee (CAAE: 73307317.8.0000.5086) and followed the Declaration of Helsinki. All participants received an explanation of the study protocol before signing the consent form.

### Statistical analysis

SPSS version 26.0 was carried out for calculation, and a *p*-value < 0.05 was adopted as statistically significant. Descriptive frequencies and *T*-test independent group tests, and chi-square comparisons were performed. Descriptive analysis of ACE-R subitems, including memory, attention, language, verbal fluency, and visuospatial skills, was also computed. Optimal sensitivity (sn) and specificity (sp) values were defined based on Youden’s index (Youden, 1950): *J*: *max* {*sn_i_* + *sp_i_* − 1}, where *i* represents the pair of coordinates on the graph. Additionally, stepwise logistic regression was performed to assess the impact of ACE-R cognitive domains on the total score. As the total and subitem scores of both ACE-R and MMSE exhibited normal curve distribution in the Kolmogorov Smirnov Test (*p* > 0.05), parametric testing was performed.

## Results

### Sociodemographic characteristics

Table 1 presents the sociodemographic and clinical characteristics of the participants in this study. Two hundred and four participants (mean age 72.65; SD 9.15) were included after initial screening (Figure 1). MCI individuals were older and had fewer years of education than controls (Table 1). Most participants were female, married (40.20%), and aged between 60 and 104 years. The mean depressive symptoms, as measured by the GDS scale, was 3.80 (SD 2.40), indicating mild symptoms (Table 1). The prevalence of MCI in our sample was 12.3%. Most participants were single homemakers (40%) and reported the most frequent comorbidities of hypertension and dyslipidemia (Table 1). The average family income of the sample was R$ 1,403.45 ($269.88), reaching an average income level in Brazil (Instituto Brasileiro de Geografia e Estatística, 2022).

**Table 1:** Socio-demographic and clinical characteristics.

Variables	Total Sample (*n* = 204)	MCI (*n* = 25; 12.25%)	Controls (*n* = 179; 87.75%)	Chi-square	Statistics	*p* value
		Number (%)			Df	
Gender						
Male	88 (43.13)	7 (28.00)	98 (54.75)			
Female	116 (56.87)	18 (72.00)	81 (45.25)	2.66	1	0.10

	Range	Mean (SD)	Mean (SD)	Mean (SD)	F	T	Df	*p* value
Socio demographic								
Age	60–104	72.65 (9.15)	79.60 (11.64)	71.68 (8.33)	9.27	−3.28	27.54	0.003
Education (years)	0–4	2.21 (1.82)	1.40 (1.63)	2.32 (1.81)	1.34	2.39	202	0.017
Household members (n)*	1–10	3.11 (1.97)	3.60 (2.16)	3.04 (1.93)	0.56	−1.33	202	0.19
Family income**	105.7–749.9	269.88 (123.75)	333.62 (158.01)	260.98 (116.43)	6.32	−2.22	27.56	0.03
Clinical symptoms								
Geriatric Depression Scale	0–13	3.80 (2.40)	5.20 (2.72)	3.59 (2.29)	0.86	−3.20	198	0.00
CVD* risk factors								
Hachinski Ischemic Score	0–15	3.96 (2.92)	4.48 (2.60)	3.89 (2.96)	0.17	−0.95	201	0.34

Clinical comorbidities	Total *N* (%)	MCI *N* (%)	Controls *N* (%)	Chi-square	Df	*p* value
Hypertension	77 (37.75)	7 (28)	70 (39.10)	2.44	2	0.30
Dyslipidemia	30 (14.70)	2 (8)	28 (15.64)	0.66	1	0.62
Diabetes	13 (6.37)	0 (0)	13 (7.26)	1.71	1	0.19
Other cardiovascular disease	21 (10.29)	1 (4)	20 (11.17)	0.92	1	0.34
Rheumatological or osteo diseases	18 (8.82)	2 (8)	16 (8.93)	0.00	1	0.98

*Number of people living in the household; ** Monthly family income in US dollars (summing all household earnings at the time of the study); CVD*, cerebrovascular risk factors; MCI, Mild Cognitive Impairment.

### Correlation between variables

The items with strongest correlation with years of education were: (a) for ACE-R, visuospatial skills (*r* = 0.497, *p* < 0.001), language (*r* = 0.363, *p* < 0.001), and verbal fluency (*r* = 0.332, *p* < 0.001); for MMSE, reading (*r* = 0.459, *p* < 0.001), writing (*r* = 0.420, *p* < 0.001) and spatial orientation (*r* = 0.359, *p* < 0.001); the level of depressive symptoms (GDS) correlated weakly with total score in MMSE (*r* = −0.171, *p* < 0.05) and ACE-R (*r* = −0.147, *p* < 0.05).

### Cognitive comparisons between groups

Tables 2, 3 depict the cognitive profile and group comparisons. Participants with MCI were older and reported fewer years of education than controls. Controls showed mean MMSE and ACE-R global scores that were significantly higher than those of MCI participants. Tables 2, 3 present the cognitive profiles of the sample, including total scores and subitems of the MMSE and ACE-R, respectively, for the control and MCI groups. Conversely, MMSE and ACE-R significantly differed between MCI and controls, except for MMSE immediate recall. Mean ACE-R subitem scores for attention, memory, verbal fluency, language, and visuospatial skills were higher in controls than MCI (Table 2).

**Table 2:** Cognitive profile of the sample – MMSE total score and subitems.

MMSE items	MCI (*n* = 25)	Controls (*n* = 179)	Statistics				Hedge’s g	95% CI
	Mean (SD)	Mean (SD)	F	T	Df	*p* value		
Time orientation	2.16 (1.43)	4.48 (0.82)	32.78	7.91	26.22	0.00	2.53	2.05–3.02
Spatial orientation	3.24 (1.48)	4.69 (0.49)	74.70	4.87	24.73	0.00	2.11	1.64–2.57
Full orientation	5.40 (2.68)	9.17 (1.04)	57.52	6.97	25.03	0.00	2.80	2.31–3.30
Immediate memory	2.52 (1.05)	2.95 (0.30)	75.60	2.04	24.57	0.05	0.94	0.51–1.36
Calculation	0.08 (0.28)	1.58 (1.67)	59.05	10.98	198.11	0.00	0.95	0.53–1.38
Memory evocation	0.96 (1.17)	2.03 (1.00)	2.62	4.92	202.00	0.00	1.05	0.61–1.47
Nomination	0.64 (0.81)	1.80 (0.56)	15.70	6.92	27.35	0.00	1.95	1.50–2.40
Repetition	0.48 (0.51)	0.92 (0.27)	60.75	4.25	25.90	0.00	1.42	0.98–1.87
Command	2.04 (1.34)	2.79 (0.56)	65.47	2.76	25.19	0.01	1.07	0.64–1.50
Reading (close eyes)	0.00 (0.00)	0.37 (0.48)	334.31	10.20	178.00	0.00	0.82	0.40–1.24
Drawing	0.00 (0.00)	0.09 (0.29)	11.95	4.18	178.00	0.00	0.33	−0.08 – 0.75
MMSE total score	12.12 (4.90)	21.99 (3.35)	4.32	9.76	27.21	0.00	2.76	2.27–3.26

MCI, Mild Cognitive Impairment; ACE-R, Addenbrooke cognitive battery – revised; MMSE, Mini Mental State Exam; CI, confidence intervals.

**Table 3:** Cognitive profile of the sample – ACE-R total score and subitems.

ACE-R items	MCI (*n* = 25)	Controls (*n* = 179)	Statistics				Hedge’s g	95% CI
	Mean (SD)	Mean (SD)	F	T	Df	*p* value		
Attention and orientation	8.08 (3.50)	13.76 (2.43)	1.97	10.33	202	0.00	2.20	1.73–2.67
Memory component I	4.00 (2.55)	8.04 (2.48)	0.06	7.60	202	0.00	1.62	1.17–2.07
Memory component II	2.28 (2.35)	5.36 (2.08)	2.75	6.82	202	0.00	1.45	1.01–1.90
Memory total	3.76 (3.54)	10.53 (3.85)	0.03	8.31	202	0.00	1.75	1.32–2.22
Verbal Fluency	1.56 (1.36)	4.47 (2.52)	9.63	8.81	51.12	0.00	1.20	0.77–1.64
Language	5.72 (3.43)	14.33 (5.11)	7.57	10.95	40.64	0.00	1.74	1.30–2.20
Viso-spatial skills	4.00 (2.55)	7.34 (3.15)	0.04	5.07	202	0.00	1.08	0.65–1.51
ACE-R total score	24.04 (9.46)	50.32 (12.44)	3.50	9.96	201	0.00	2.16	1.70–2.63

MCI, Mild Cognitive Impairment; ACE-R, Addenbrooke’s cognitive battery – revised.

Memory component I: immediate recall, retrograde and anterograde; Memory component II: late recall e recognition; Memory total: composite score. CI – confidence intervals.

### Logistic regression analysis

The R-square value indicates that the three predictor variables explain about 94.9% of the variance in ACE-R. The *β* values indicate the relative influence of the entered variables, that is, language has the most significant influence on ACE-R total score (*β* = 0.54, *t*(199) = 27.11, *p* < 0.001), followed by memory (*β* = 0.37, *t*(199) = 17.52, *p* < 0.001), and then verbal fluency (*β* = 0.26, *t*(199) = 14.16, *p* < 0.001). The direction of influence for all three is positive (Table 4).

**Table 4:** Linear regression models of predictable variables for ACE-R performance.

Models	R^2^	ΔR^2^	Standard error	R square change	F	*p* value
1. Language	0.76	0.76	7.30	0.76	628.55	<0.001
2. Language, memory	0.90	0.90	4.75	0.14	880.42	<0.001
3. Language, memory, verbal fluency	0.95	0.95	3.36	0.05	1239.67	<0.001
4. Language, memory, verbal fluency, attention and orientation	0.97	0.97	2.36	0.03	1933.83	<0.001

### Receiver operating characteristic (ROC) curve analysis for group comparisons

**Figure 2:**
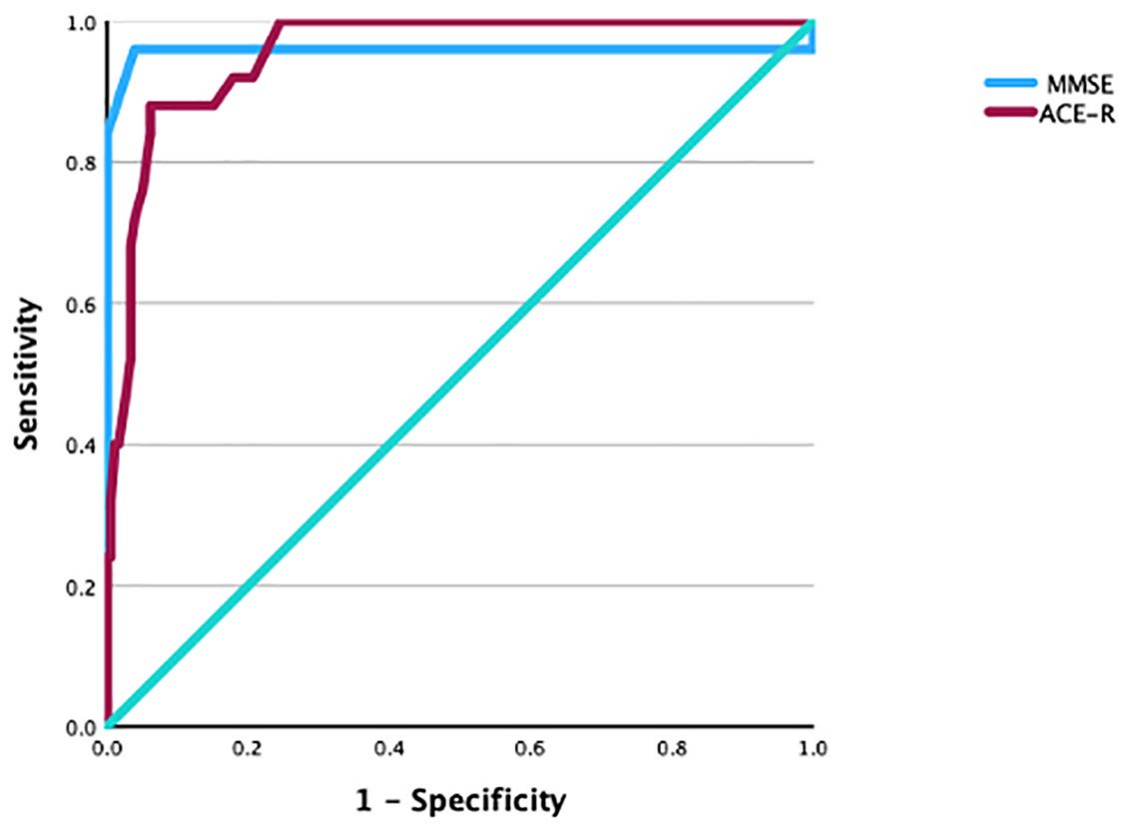
ROC curve of MMSE and ACE-R (for statistical details, see results).

We assessed sn and sp. for the MMSE and ACE-R using receiver operating characteristic (ROC) curves (Figure 2).

When we compared controls versus MCI, the area under the ROC curve (AUC) for the MMSE was 0.96, which is considered excellent using Meyers’ scale (Meyers et al., 2009); the MMSE showed 0.96 sn and 0.75 sp. (95% CI 0.88–1.00; *p* ≤ 0.001) for a cutoff score of 19.5 (Figure 2). For the ACE-R, the AUC was 0.96, which is considered excellent (Meyers et al., 2009); it showed 1.00 sn and 0.75 sp. (95% CI 0.92–0.99; *p* ≤ 0.001) for a cutoff score of 40.5 (Figure 2).

## Discussion

Our study assessed the cognitive performance of a rural sample of Quilombola population with low educational attainment, comprising nondementia individuals, including cognitively healthy and MCI participants. The general sociodemographic characteristics were described, and the accuracy of two screening tools for cognitive disorders, the ACE-R and MMSE, was examined. The diagnostic accuracy of the MMSE and ACE-R for MCI and healthy controls was substantially lower than that reported in Brazilian studies with low schooling subjects living in urban areas. Language and verbal fluency were the subitems with the strongest correlation with better performance on the ACE-R. Our findings suggest cutoff scores for interpreting ACE-R in a non-dementia sample with low educational attainment, taking into account factors related to the ecological context, particularly living in hinterland areas. Such results can help improve diagnostic accuracy for diagnosing MCI and healthy controls and ease early therapeutic interventions.

Overall, mean scores among adults with MCI from the IQUIBEQ project are considerably lower than those described in the literature. International studies, such as those by Alexopoulos et al., reported higher mean ACE-R scores for cognitive performance (controls: 90.37 ± 4.99; MCI: 81.34 ± 9.09). In Brazil, three studies by Caramelli et al. investigated ACE-R performance among Brazilian patients (Amaral-Carvalho and Caramelli, 2012; Carvalho et al., 2017; Carvalho and Caramelli, 2007; Amaral-Carvalho and Caramelli, 2022). One of them, conducted with 144 healthy older adults, found higher ACE-R scores among adults aged 60 to 79 (79.5 ± 0.86) compared to our study (50.32 ± 12.44). Furthermore, the outlined study (Amaral-Carvalho and Caramelli, 2012) included more educated participants than our study (mean years of education) for the group with 4–7 years of education (4.73 vs. 2.3). A comparison of our results with a study from Passinho et al. in 2024 with an urban sample of outpatient subjects and low schooling, showed lower scores for all ACE-R subitems: memory (10.53 vs. 16.42), verbal fluency (4.47 vs. 7.12), language (14.33 vs. 18.8), visuospatial skills (7.34 vs. 10.57) and attention/orientation (13.76 vs. 14.10) (Amaral-Carvalho and Caramelli, 2012); in addition, MMSE total scores for healthy controls in our study were also lower (21.99 vs. 25.68) (Tavares-Júnior et al., 2021; Passinho et al., 2024).

Additional Brazilian studies involving rural populations have yielded heterogeneous findings. One study with a small sample size (*n* = 34) conducted by Rigo et al. (2010) in South Brazil reported higher MMSE values for both illiterate individuals (23.60 ± 2.30) and subjects with 1–3 years of schooling (24.40 ± 2.7) when compared to our sample (21.99 ± 3.35). Another cross-sectional study (*n* = 806 individuals) by Martins et al. (2016) in Pelotas reported similar values for subjects with 0–3 years of schooling (21.27 ± 0.08); however, neither Rigo nor Martins explored cognitive status (MCI or CIND) (Rigo et al., 2010; Martins et al., 2016). Our findings thus provide additional evidence to the literature, highlighting the heterogeneity of the population with low educational attainment, particularly those of the Brazilian hinterland.

In our results, language and verbal fluency were found to be predictors of global cognitive performance in the ACE-R. These findings also highlighted the importance of expanded cognitive batteries (such as the ACE-R) of verbal and language components. One hypothesis is that older adults with low educational attainment would be more likely to recruit language and executive skills as compensatory resources than better-educated participants (Bertola et al., 2019). Our findings also align with previous evidence, in which greater cognitive performance on verbal knowledge may be related to learning of verbal information, while better executive function may facilitate efficient memory retrieval of the stored material (Bouazzaoui et al., 2013; Rast, 2011); Indeed, a substantial proportion of healthy controls in the IQUIBEQ sample presented preserved episodic memory, supporting previous evidence showing memory-protective factors may differ across older adults with distinct educational backgrounds, and the need to evaluate a broader range of potential resilience factors for older adults with low schooling (Bertola et al., 2019).

Furthermore, lower visuospatial performance was strongly influenced by education, a finding already reported by Okada (2021). One study showed higher visuospatial skills as a strong predictor of episodic memory performance for individuals with middle education attainment (Bertola et al., 2019). Visuospatial and executive deficits may often signify underdiagnosed subtypes of cognitive disorders such as Lewy bodies and Parkinson’s disease (McKeith et al., 2017), alongside memory impairment, the use of these tools for the purpose of populational screening may be desirable (Breton et al., 2019). Other screening instruments for the general population, such as the Brief Cognitive Screening Battery (Fichman-Charchat et al., 2015) and RUDAS (de Araujo et al., 2018), employ, respectively, the clock drawing test and cube copy; however, it is still unclear whether the potential advantage of these items over other cognitive batteries in the screening of MCI in rural populations.

Our findings provide additional evidence indicating that short versions of ACE-R, such as the Mini-ACE-R proposed by Miranda et al. (2018) and Okada (2021), offer higher accuracy than the MMSE and should be considered an alternative for cognitive screening of subjects with limited schooling. In addition, Amaral-Carvalho et al. (2024) proposed a short version of ACE-R to reduce educational bias through the Mokken Scale technique, which includes orientation in time and memory recognition. Their results enhanced the accuracy of discriminating between controls and MCI (Amaral-Carvalho et al., 2024).

The IQUIBEQ results shed light on the distinct cognitive trajectories between individuals living in rural and urban areas. Generally, individuals in hinterland areas have limited access to formal education, healthcare, and basic sanitation. Despite this lack of resources, cognitive performance strategies may rely on compensatory strategies, such as the quality of education, which is deemed more important than the number of years of schooling (Sisco et al., 2015; Manly et al., 2003). Additionally, the role of fluid and crystalline intelligence, which make up the general intelligence factor defined by Cattell (1971), in which cognitive skills may be enhanced regardless of formal education, becomes a relevant factor in cognitive reserve, as pointed out in the literature (Sanginabadi, 2020; Tucker-Drob et al., 2009). Finally, the ecological validity of cognitive screening tests should be considered when establishing the accuracy of the tests and generalizing the findings (Pinto et al., 2025).

Health public policies that address the needs and environmental aspects of communities may be more effectively driven when the cognitive profile is better understood (Pinto et al., 2025). Our study also confirms the importance of stratifying norms for elderlies with fewer than 4 years of education. Our results are consistent with previous studies indicating that the ACE-R remains an effective screening tool for individuals with lower levels of education (Breton et al., 2019; McKeith et al., 2017; Amaral-Carvalho et al., 2024), provided that education-adjusted values and geographical context are used as the reference for average performance. Additionally, using shortened versions of the ACE-R could help address the limitations of this instrument, especially in evaluating visuospatial abilities.

The current study found a relatively low prevalence of MCI at 12.5%. In comparison, other Brazilian studies report varying prevalence rates. For instance, previous studies have estimated rates ranging from 12.5 to 17.5% in the population aged 60 years or older (Ministério da Saúde, 2024a). Notably, studies that utilize clinical diagnoses tend to present higher estimates of dementia. Two studies based on algorithms to define dementia cases within the ELSI-Brazil cohort reported national prevalences of 5.8 and 6.3%, both significantly lower than those from clinical diagnosis. Additionally, a recent population-based study, the ELSI project, found an estimated prevalence of CIND of 9.4% among illiterates across various Brazilian regions (Bertola et al., 2023).

To the best of our knowledge, the IQUIBEQ project is the first study to assess the accuracy properties of a global cognitive screening tool in MCI in rural Quilombola communities in Brazil. One of the strengths of our study was the recruitment of individuals through local community agents, which helped minimize participant losses during the recruitment process. Additionally, the involvement of local stakeholders fostered a collaborative relationship with the research team, a connection that has been established since the inception of the current project (Costa et al., 2021).

Our study has some limitations that deserve consideration. First, we cannot establish cause-and-effect relationships from cross-sectional data, and follow-up studies would be required to confirm our hypothesis. Second, the concept of MCI also includes multi-domain subtypes, which were not included in our sample study, even though some studies have questioned the validity of these and other subdomains of MCI (Klekociuk and Summers, 2014). Additionally, concerning the MCI group, its relatively low prevalence and the etiological investigation of cases could not be established, primarily due to the limitations of biomarkers, such as genetic tests, plasma amyloid tests, and PET-CT scans. Third, the participant’s level of education was self-reported and, unlike previous investigations, we have not stratified educational years for the statistical analysis (Bertola et al., 2019). We have acknowledged, in fact, the intrinsic limitations of using years of education as a stratifying variable in our selected sample of rural Quilombola participants. These limitations are primarily due to the low quality and precarious access to formal education, as well as the limited reliability and reduced statistical power of such analysis. Indeed, most studies need to consider the quality of education while studying adults with low levels of education, thus preventing an underestimation of the effect of this variable. Recent research has investigated other variables, including language skills, vocabulary, cognitive reserve, knowledge acquired throughout life, abstraction ability, and formal-logical operational capacity, which are deemed more sensitive to establishing educational status (Bertola et al., 2019). Finally, there is also the bias of external validation or extrapolation of data to rural populations in other geographic areas of Brazil, another factor that limits our findings (Ministério da Saúde, 2024b; Simon et al., 2024).

## Conclusion

Our study assessed ACE-R and MMSE performance in older healthy controls and MCI subjects in a rural Quilombola community. Average scores for healthy aging were considerably lower than those reported in prior Brazilian studies of rural and urban populations, which employed similar methodologies. The analysis of ACE-R diagnostic accuracy between non-dementia groups also evidenced lower cutoff scores compared to benchmark Brazilian studies. Our findings highlight the need for more studies on cognitive changes in Brazil’s older adults with low education levels and MCI living in rural areas. The ecological value, cultural characteristics, and heterogeneity of illiterate groups should be considered in the analysis. This study provides additional evidence to support screening approaches and strategies, particularly in primary care and health community services, on how to achieve early diagnosis, optimizing therapeutic intervention for subjects with low schooling and cognitive decline at risk for conversion to dementia. Our results also encourage upcoming studies on the early screening of cognitive decline and at risk for dementia in Quilombola communities and other underserved populations.

## Data Availability

The datasets presented in this study can be found in online repositories. The names of the repository/repositories and accession number(s) can be found in the article/Supplementary material.
