# Supplementary material for: Accuracy of the revised Addenbrooke Cognitive Examination (ACE-R) and Mini-Mental (MMSE) in a Quilombola community with low education attainment: results of a cross-sectional study
Source: Front Dement. 2026 Feb 2;4:1673362. doi: 10.3389/frdem.2025.1673362 (PMC12907204; doi:10.3389/frdem.2025.1673362)
Supplement: Supplementary file 1 [file Table_1.DOCX]

**Supplementary material**

**Table 1.** Socio demographic characteristics of individuals in the Quilombola area

| **Socio-demographic variables** | **Total (n = 204)** | **MCI (n = 25)** | **Controls (N = 179)** |
| --- | --- | --- | --- |
| **Personal** | **Number (%)** | | |
| *Gender* |  |  |  |
| Male | 88 (43.13) | 7 (28.00) | 98 (54.75) |
| Female | 116 (56.87) | 18 (72.00) | 81 (45.25) |
| *Marital Status* |  |  |  |
| Married/stable union | 82 (40.20) | 5 (20.00) | 77 (43.02) |
| Divorced | 17 (8.33) | 1 (4.00) | 16 (8.93) |
| Widowed | 45 (22.06) | 9 (36.00) | 36 (20.11) |
| Single | 60 (29.41) | 10 (40.00) | 50 (27.93) |
| **Household characteristics** |  |  |  |
| *Predominant material of external walls* |  |  |  |
| Masonry with coating | 147 (72.06) | 14 (56.00) | 133 (74.30) |
| Masonry without coating | 37 (18.14) | 6 (24.00) | 31 (17.32) |
| Uncoated wattle and daub | 12 (5.88) | 4 (16.00) | 8 (4.47) |
| Coated wattle and daub | 8 (3.92) | 1 (4.00) | 7 (3.91) |
| *Source of electricity* |  |  |  |
| General power grid | 201 (98.53) | 25 (100.00) | 176 (98.32) |
| Other sources (generator, solar panel, etc.) | 3 (1.47) | 0 (0.00) | 3 (1.68) |
| *Worked in the last month for at least one hour in any paid activity* | | |  |
| Yes | 16 (7.84) | 2 (8.00) | 14 (7.82) |
| No | 188 (92.16) | 23 (92.00) | 165 (92.18) |
| *Water supply* |  |  |  |
| General distribution network | 54 (26.47) | 3 (12.00) | 51 (28.49) |
| Well or spring on the property | 118 (57.84) | 16 (64.00) | 102 (56.98) |
| Well or spring outside the property | 30 (14.71) | 6 (24.00) | 24 (13.41) |
| Water truck | 1 (0.49) | 0 (0.00) | 1 (0.56) |
| Rainwater stored in a cistern | 1 (0.49) | 0 (0.00) | 1 (0.56) |
| **Health Variables** |  |  |  |
| *Type of drinking water* |  |  |  |
| Filtered | 140 (68.63) | 14 (56.00) | 126 (70.39) |
| Boiled | 7 (3.43) | 0 (0.00) | 7 (3.91) |
| Industrialized mineral | 10 (4,90) | 0 (0.00) | 10 (5.59) |
| Untreated | 8 (3.92) | 2 (8.00) | 6 (3.35) |
| Other | 39 (19.12) | 9 (36.00) | 30 (16.76) |
| *Participation in group sports or artistic activities in the last 12 months* | | | |
| More than once a week | 3 (1.47) | 0 (0.00) | 3 (1.68) |
| Once a week | 2 (0.98) | 0 (0.00) | 2 (1.12) |
| 2 to 3 times a month | 2 (0,98) | 0 (0.00) | 2 (1.12) |
| A few times a year | 17 (8.33) | 0 (0.00) | 16 (8.94) |
| Once a year | 1 (0.49) | 1 (4.00) | 1 (0.56) |
| Never | 179 (87.75) | 24 (96.00) | 155 (86.59) |
| *Engagement in any type of physical exercise or sports in the last three months* | | | |
| 0 | 1 (0.49) | 0 (0.00) | 1 (0.56) |
| Yes | 20 (9.80) | 1 (4.00) | 19 (10.61) |
| No | 183 (89.71) | 24 (96.00) | 159 (88.83) |
| *Diagnosis of any chronic disease (physical or mental) or long-term illness lasting more than 6 months* | | | |
| Yes | 130 (63.73) | 18 (72.00) | 112 (62.57) |
| No | 74 (36.27) | 7 (28.00) | 67 (37.43) |

Abbreviations: MCI – mild cognitive impairment

**Table 2.** Comparative analysis of group performance and accuracy metrics on the ACE-R (a) and MMSE (b), in reference to findings reported in the existing literature ^τ^

a)

| **Group comparison** | **AUC** | **Optimal Cutoff** | **Sensitivity** | **Specificity** | **P value** | **CI** | **LR +** | **LR -** |
| --- | --- | --- | --- | --- | --- | --- | --- | --- |
| Controls vs. naMCI^τ^ | 0.92 | <59.5 | 0.95 | 0.75 | <0.00 | 0.86-0.97 | 3.80 | 0.07 |
| Controls vs. aMCI^τ^ | 0.92 | <53 | 0.78 | 0.92 | <0.00 | 0.86-0.99 | 9.75 | 0.24 |
| naMCI vs. aMCI^τ^ | 0.58 | <48.5 | 0.56 | 0.64 | <0.00 | 0.24-0.61 | 1.56 | 0.69 |
| Controls vs. MCI* | 0.96 | <40.50 | 1.00 | 0.75 | <0.00 | 0.92 - 0.99 | 4.00 | 0.00 |

Abbreviations: ^τ^ Passinho et al., 2024; * current study; AUC – area under the curve; MCI – Mild Cognitive Impairment; CI – confidence interval; LR - likelihood ratio; naMCI – non amnestic MCI; aMCI – amnestic MCI

b)

| **Group Comparison** | **AUC** | **Optimal Cutoff** | **Sensitivity** | **Specificity** | **P value** | **CI** | **LR +** | **LR -** |
| --- | --- | --- | --- | --- | --- | --- | --- | --- |
| Controls vs. naMCI^τ^ | 0.93 | <24.5 | 0.95 | 0.85 | <0.00 | 0.87-0.98 | 6.33 | 0.06 |
| Controls vs. aMCI^τ^ | 0.86 | <24.5 | 0.83 | 0.85 | <0.00 | 0.77-0.96 | 5.53 | 0.20 |
| naMCI vs. aMCI^τ^ | 0.61 | ≤22.5 | 0.73 | 0.39 | <0.00 | 0.43-0.79 | 1.20 | 0.69 |
| Controls vs. MCI^*^ | 0.96 | <19.50 | 0.96 | 0.75 | <0.00 | 0.88 - 1.00 | 3.84 | 0.05 |

Abbreviations: ^τ^ Passinho et al., 2024; * current study; AUC – area under the curve; MCI – Mild Cognitive Impairment; CI– confidence interval; LR - likelihood ratio; naMCI – non amnestic MCI; aMCI – amnestic MCI;

**Table 3.** cutoff scores of ACE-R (a) and MMSE (b)

a)

| **ACE-R** | **Sensitivity** | **Specificity** | **LR+** | **LR-** | **PPV** | **NPV** |
| --- | --- | --- | --- | --- | --- | --- |
| 36.5 | 0.88 | 0.88 | 7.33 | 0.14 | 0.51 | 0.98 |
| 38.5 | 0.92 | 0.82 | 5.11 | 0.10 | 0.42 | 0.99 |
| **40.5** | **1.00** | **0.75** | **4.00** | **0.00** | **0.36** | **1.00** |
| 44.5 | 1.00 | 0.66 | 2.94 | 0.00 | 0.29 | 1.00 |
| 46.5 | 1.00 | 0.61 | 2.56 | 0.00 | 0.26 | 1.00 |
| 48.5 | 1.00 | 0.55 | 2.22 | 0.00 | 0.24 | 1.00 |
| 50.5 | 1.00 | 0.50 | 2.00 | 0.00 | 0.22 | 1.00 |
| 52.5 | 1.00 | 0.44 | 1.79 | 0.00 | 0.20 | 1.00 |
| 54.5 | 1.00 | 0.39 | 1.64 | 0.00 | 0.19 | 1.00 |
| 56.5 | 1.00 | 0.31 | 1.45 | 0.00 | 0.17 | 1.00 |
| 58.5 | 1.00 | 0.24 | 1.32 | 0.00 | 0.16 | 1.00 |
| 60.5 | 1.00 | 0.20 | 1.25 | 0.00 | 0.15 | 1.00 |
| 64.5 | 1.00 | 0.12 | 1.14 | 0.00 | 0.14 | 1.00 |
| 67.5 | 1.00 | 0.09 | 1.10 | 0.00 | 0.13 | 1.00 |
| 70.5 | 1.00 | 0.05 | 1.05 | 0.00 | 0.13 | 1.00 |
| 75.5 | 1.00 | 0.02 | 1.02 | 0.00 | 0.12 | 1.00 |
| 79 | 1.00 | 0.01 | 1.01 | 0.00 | 0.12 | 1.00 |
| 86 | 1.00 | 0.00 | 1.00 | - | 0.12 | - |

Abbreviations: ACE-R- Addenbrooke cognitive battery – revised; LR - LR - likelihood ratio, PPV – Positive Predictive Value, NPV- Negative Predictive Value

b)

| **MMSE** | **Sensitivity** | **Specificity** | **LR+** | **LR-** | **PPV** | **NPV** |
| --- | --- | --- | --- | --- | --- | --- |
| 15.5 | 0.84 | 1.00 | - | 0.16 | 1.00 | 0.98 |
| 17.5 | 0.96 | 0.89 | 8.73 | 0.04 | 0.55 | 0.99 |
| 19.5 | 0.96 | 0.75 | 3.84 | 0.05 | 0.35 | 0.99 |
| 21.5 | 0.96 | 0.57 | 2.23 | 0.07 | 0.24 | 0.99 |
| 23.5 | 0.96 | 0.34 | 1.45 | 0.12 | 0.17 | 0.98 |
| 25.5 | 0.96 | 0.15 | 1.13 | 0.27 | 0.14 | 0.96 |
| 27.5 | 0.96 | 0.06 | 1.02 | 0.67 | 0.12 | 0.91 |

Abbreviations: MMSE – Mini Mental State Exam; LR - LR - likelihood ratio, PPV – Positive Predictive Value, NPV- Negative Predictive Value
